# Differential ketogenic diet-induced shift in CSF lipid/carbohydrate metabolome of pediatric epilepsy patients with optimal vs. no anticonvulsant response: a pilot study

**DOI:** 10.1186/s12986-020-00524-1

**Published:** 2021-03-01

**Authors:** Susan A. Masino, David N. Ruskin, Natalie R. Freedgood, Marie Lindefeldt, Maria Dahlin

**Affiliations:** 1 Department of Psychology and Neuroscience Program, Trinity College, Hartford, CT 06106 USA; 2 Neuropediatric Department, Astrid Lindgren Children’s Hospital, Karolinska Hospital, Stockholm, Sweden

**Keywords:** Acetoacetate, Anticonvulsant, β-hydroxybutyrate, Cerebrospinal fluid, Glucose, Ketogenic diet, Pediatric epilepsy

## Abstract

**Background:**

The low carbohydrate, high fat ketogenic diet can be an effective anticonvulsant treatment in some pediatric patients with pharmacoresistant epilepsy. Its mechanism(s) of action, however, remain uncertain. Direct sampling of cerebrospinal fluid before and during metabolic therapy may reveal key changes associated with differential clinical outcomes. We characterized the relationship between seizure responsiveness and changes in lipid and carbohydrate metabolites.

**Methods:**

We performed metabolomic analysis of cerebrospinal fluid samples taken before and during ketogenic diet treatment in patients with optimal response (100% seizure remission) and patients with no response (no seizure improvement) to search for differential diet effects in hallmark metabolic compounds in these two groups. Optimal responders and non-responders were similar in age range and included males and females. Seizure types and the etiologies or syndromes of epilepsy varied but did not appear to differ systematically between responders and non-responders.

**Results:**

Analysis showed a strong effect of ketogenic diet treatment on the cerebrospinal fluid metabolome. Longitudinal and between-subjects analyses revealed that many lipids and carbohydrates were changed significantly by ketogenic diet, with changes typically being of larger magnitude in responders. Notably, responders had more robust changes in glucose and the ketone bodies β-hydroxybutyrate and acetoacetate than non-responders; conversely, non-responders had significant increases in fructose and sorbose, which did not occur in responders.

**Conclusions:**

The data suggest that a differential and stronger metabolic response to the ketogenic diet may predict a better anticonvulsant response, and such variability is likely due to inherent biological factors of individual patients. Strategies to boost the metabolic response may be beneficial.

## Introduction

Epilepsy affects 1 in 26 people across the lifespan [1], and 25–30% of patients suffer uncontrolled seizures in spite of pharmacotherapy [2]. In addition, the incidence of side effects of anticonvulsants is high, and can lead to cessation of medications [3, 4]. Ketogenic diet (KD) is a metabolic therapy that employs a high-fat, low-carbohydrate, moderate-protein formulation developed initially to mimic the metabolic state of fasting [5, 6]: it is established as anticonvulsant [7], antiepileptic [8], neuroprotective [9], and particularly useful in some specific diagnoses [10] and in refractory epilepsy [11, 12].

Fasting and adhering to a KD each produce hallmark changes in blood ketone bodies (increased) and glucose (decreased) but it has remained unclear how these changes relate to beneficial effects. For instance, blood ketone body levels do not always correlate with anticonvulsant effects [13], and in general anticonvulsant benefits of ketones versus glucose in clinical and laboratory work is mixed and still under active investigation and debate [14]. Furthermore, mechanistic links between these metabolic blood chemistry changes and anticonvulsant effects are unclear. Clearly many systemic changes occur with dietary therapy, making it difficult to sort out key mechanisms of efficacy. To date a number of studies have examined changes in blood metabolites aside from ketone bodies and glucose, typically examining a limited number of predetermined compounds to address specific hypotheses [15–21]. While cerebrospinal fluid (CSF) is significantly more difficult to collect than blood, analysis of CSF metabolites may be more relevant to KD mechanisms in epilepsy [22–25].

Metabolomics is a liquid chromatography/gas chromatography/mass spectroscopy-based technique for quantifying all detectable small molecule metabolites, such as carbohydrates, small lipids, amino acids, etc. and has been used to describe biological phenotypes in epilepsy [26] and other neurological disorders [27] as well as dietary treatments [28]. Extant metabolomics studies of serum and ketogenic diet have described compensatory adaptations in amino acid metabolism [29] and correlations between metabolites and fecal microbiota [30] in experimental rodents. In general, metabolomic analysis of CSF could be a powerful tool for discovering KD-related mechanisms. Here we present novel data based on a set of CSF samples collected twice from pediatric patients with refractory seizures – once before KD treatment and once during KD treatment. This experimental design enables each patient to serve as their own control, and thus the ability to compare baselines and changes in the metabolome to the observed clinical response. A metabolomic analysis of carbohydrates and lipids could relate the biochemical effects of the KD to the magnitude of the clinical (anticonvulsant) response, and enables a review of predictors of KD efficacy.

## Methods

### Ethics

The study was approved by the Ethics Committee of the Karolinska Hospital and the informed consent of the parents and, when possible, patients, was obtained.

### Patients

Patients have been described in detail previously [22, 23]. Briefly, the study was part of a larger prospective open trial on the efficacy and safety of the KD in children with epilepsy. The patients were enrolled consecutively as they attended the Epilepsy Outpatient Clinic and the decision to start the KD was made.

Inclusion criteria were an age of 1–18 years, pharmacologically refractory epilepsy with a prior trial of at least three antiepileptic drugs, consideration of epilepsy surgery, no medical contraindications, family and patient able to cooperate in a KD trial, and approval for performing two lumbar punctures before and during KD. Parents and other caregivers made daily notes on the number and type(s) of seizures on seizure calendars routinely used in the clinic. Parental reports were compared with the observations of seizure type and frequency made by the epilepsy nurses during hospitalization. A subgroup of 27 children were selected based on the most differentiated clinical outcome – namely those observed as seizure-free seizure and those with no observed improvement. Five of the patients became seizure-free and were classified as having “optimal response” and five patients had no detectable seizure reduction and were classified as having “no response” (see Table 1). In these ten included children metabolomics analysis with a focus on changes in carbohydrates and lipids. These patients all had variable levels of intellectual disability except for one who had autism spectrum disorder; patients were a mix of verbal and non-verbal.

**Table 1:** Patient demographics, sorted by clinical response

**Characteristics of 5 patients with optimal seizure reduction (no detected seizures)**						
sex	age	Epilepsy type(s)	Seizure type(s)	Etiology/syndrome	AEDs	Diet ratio
M	4.5	generalized	Myoclonic, generalized T/C	Doose syndrome	VPA, CZP	4:1
F	4.8	generalized	Myoclonic, tonic, absence	L-G syndrome	CLB	3:1
M	5.1	generalized	Myoclonic, tonic	Down’s syndrome	VPA, LTG	4:1
F	5.8	generalized	Myoclonic, generalized T/C	Cortical dysplasia (polymicrogyria)	none	3:1
F	9.0	focal	Focal without impaired awareness	Cortical dysplasia	PRI, VPA	4:1
Mean:	5.8					
**Characteristics of 5 patients with no observed change in seizures**						
M	4.0	generalized	Myoclonic, tonic, epileptic spasms	Inverted duplicated chromosome 15, L-G syndrome	LTG, TPM	4:1
M	6.0	generalized	Epileptic spasms, generalized T/C	L-G syndrome	VPA, LTG	3:1
M	6.3	generalized	Myoclonic, absence, generalized T/C	Infantile spasms, L-G syndrome	VPA, CLB	4:1
F	6.7	generalized	Generalized T/C	Unknown	LTG, PB, TPM	4:1
M	10.1	generalized	Myoclonic, atonic	Agenesi corpus callosum, other CNS malformations	VPA, CZP	4:1
Mean:	6.6					

Seizure types classified according to the International League Against Epilepsy (ILAE) classification [31]. “Diet ratio” indicates the (fat:(protein + carbohydrate)) ratio of the KD at time of 2nd lumbar puncture. *Abbreviations*: *AED* antiepileptic drug, *T/C* tonic/clonic, *L-G* Lennox-Gastaux syndrome, *VPA* valproic acid, *CZP* clonazepam, *CLB* clobazam, *LTG* lamotrigine, *PRI* primidone, *TPM* topiramate, *PB* phenobarbital

### Study design

This pre-KD/during KD retrospective design, performed between 1998 and 2002 at the Neuropediatric Department of the Astrid Lindgren Children’s Hospital, (Karolinska Hospital), has been described in detail previously [22, 23]. To initiate the diet, patients were hospitalized during a 4–5 day stay. The 1st CSF sampling was collected via lumbar puncture obtained immediately before diet start and the 2nd CSF sampling was performed during a 2 day inpatient stay about 3 months after KD initiation when major fine-tuning adjustments of the diet were made. During the 2nd sampling the patients were on 3:1 or 4:1 KD ratio (Table 1). All lumbar punctures were performed in the morning, around 8 AM, soon after breakfast. During both lumbar punctures, four mL CSF was collected: samples with visible blood content were not used. Aliquots were immediately cold-centrifuged, supernatant collected, frozen, and stored at − 70 °C. Prior studies have shown that metabolites stored at this temperature are expected to remain stable for decades [32, 33]. Serum β-hydroxybutyrate (β-HB) levels were measured the same morning before breakfast of the 2nd lumbar puncture and analyzed consecutively.

The diet followed a standardized protocol for the classic KD, which is a slightly modified version of the protocol of the Johns Hopkins Hospital [34]. Most patients were started at a 4:1 (fats:(proteins + carbohydrates)) ratio. A minimum of 1 g/kg body weight per day of protein was used. At diet start supplements of vitamins and minerals were given to all patients as well as carnitine (100 mg/kg body weight per day). All calculations of the amount of calories and the nutrient composition of the menus of the diet were made for the individual child by a specially trained dietician.

The parents were educated on all aspects of the diet by a specialized dietician, neuropediatrician, and keto nurse during a 4–5 days inpatient stay at diet start. The parents were carefully instructed on the importance of being accurate in preparing the menus of the diet, and on keeping detailed diaries. Dietary compliance was monitored by daily testing of urine ketones at home by parents with data reported to nurses, and also by blood testing for β-hydroxybutyrate during frequent visits with the diet team consisting of nurse, dietician and neuropediatrician. Members of the team had frequent (sometimes daily) telephone consultations with parents on how to manage the diet. Also, diaries were discussed with the team during clinic visits at 1 and 3 months of treatment. Medications were not changed during the study, with the exception of slight decreases in some children.

### Metabolomics

Frozen CSF samples (20 total) were sent to Metabolon (Durham, North Carolina) for metabolomics analysis in 2014. Samples were prepared and extracted. Internal standards were added to every analyzed sample as an internal quality control; samples were split into fractions for ultra-high performance liquid chromatography-tandem mass spectrometry (UPLC-MS/MS) with positive ionization, UPLC-MS/MS with negative ionization, and gas chromatography–mass spectrometry (GC-MS). The UPLC-MS/MS platform utilizes Waters Acquity UPLC with Waters UPLC BEH C18 or BEH Amide 2.1 × 100 mm, 1.7 μm columns and a ThermoScientific Q-Exactive high resolution/accurate mass spectrometer interfaced with a heated electrospray ionization (HESI-II) source and Orbitrap mass analyzer operated at 35,000 mass resolution. The GC-MS platform utilizes a Thermo-Finnigan Trace DSQ fast-scanning single-quadrupole MS using electron impact ionization operated at unit mass resolving power (scan range 50–750 m/z), with a 5% diphenyl/95% dimethyl polysiloxane fused silica column (20 m × 0.18 mm ID; 0.18 um film thickness) with helium as carrier gas and a temperature ramp from 60° to 340 °C in a 17.5 min period. Metabolites were identified by automated comparison of the ion features in the experimental samples to a reference library of chemical standard entries that include retention time, molecular weight (m/z), preferred adducts, and in-source fragments as well as associated MS spectra. Identification of known chemical entities was based on comparison to metabolomic library entries of ~ 4500 purified standards. Raw data (ion counts) for each metabolite were scaled so that the median equaled one; missing values (non-detections) were imputed with half the minimum value. Statistical analysis (performed by Metabolon) included ANOVA, and paired and unpaired t-tests as appropriate. False discovery rate (q) was calculated for multiple comparisons: here we consider q < 0.10 to indicate high confidence in a result, and 0.10 < q < 0.20 to indicate moderate confidence.

## Results

The patient group included all five children with 100% seizure reduction (“optimal response”) and all five patients with no seizure reduction; demographics of the patient group are given in Table 1. Patients with optimal seizure response and no observed seizure remission to KD treatment had similar age ranges and included both males and females. Seizure types and the etiology of the epilepsy or epilepsy syndrome varied and did not appear to differ systematically between patients with an optimal vs. no observable clinical response. Similarly, anticonvulsant drug treatment did not appear to differ systematically between the two groups, nor did KD ratio. Statistical analysis was not performed on these factors due to variability, overlap in diagnostic categories and small sample size.

In CSF samples from these patients, 271 metabolites were identified. We focused on analyzing CSF compounds in the lipid and carbohydrate families (49 and 25 compounds detected, respectively. In the lipid family 25/49 compounds changed significantly (comparing baseline to during KD) in patients with an optimal vs. no observable clinical response; in the carbohydrate family, 15/25 changed significantly in optimal or no observable clinical response (Table 2). Considering both families together, there was a strong trend (*p* = 0.066) for more compounds to change baseline-to-KD in patients with an optimal vs. no observable clinical response.

**Table 2:** Ketogenic diet-induced changes in lipids

		During diet/Prediet	
Compound Family	Compound	optimal response	no response
Ketone Bodies	1,2-propanediol	29.16***	7.47**
	Acetoacetate	130.8***	67.5***
	β-hydroxybutyrate	175.5***	57.4***
Acyl Carnitines	Propionyl-carnitine	1.50*	1.48
	Acetyl-carnitine	5.19***	3.89*
	Butyryl-carnitine	5.51**	2.40
	Octanoyl-carnitine	9.43***	5.44
	Hexanoyl-carnitine	16.09***	4.73**
	3-hydroxybutyryl-carnitine	23.63***	15.84**
Carnitine Metabolism	Carnitine	0.99	1.19
	Deoxycarnitine	2.00	1.35
	3-dehydrocarnitine	61.96***	108.5***
Monohydroxy Fatty Acids	2-hydroxyhexanoate	1.28	2.10
	3-hydroxypropanoate	2.35	1.46
	γ-hydroxybutyrate	3.40***	2.06**
	5-hydroxyhexanoate	6.48*	2.29*
	3-hydroxyoctanoate	10.35***	3.48***
	3-hydroxysebacate	22.12**	6.70
Medium Chain Fatty Acids	Nonanoic acid	0.97	1.19
	Dodecanoic acid	1.05	0.98
	Decanoic acid	1.14*	1.12
	Octanoic acid	1.90	1.15
	Heptanoic acid	2.24	0.73
	Hexanoic acid	3.29	1.49
Phospholipid Metabolism	Choline	0.96	0.94
	Phosphoethanolamine	1.02	1.26
	Choline phosphate	1.47	2.50
	Trimethylamine N-oxide	715.1**	375.3**
Amide Fatty Acid	Oleamide	0.99	16.48
Dicarboxylate Fatty Acids	2-hydroxyglutarate	2.05	1.07
	CMPF	8.81*	2.61
Triglyceride Metabolism	Glycerol 3-phosphate	0.82**	0.86
	Glycerol	1.22	0.89
Lysophospholipids	1-oleoyl-GPC (18:1)	0.46*	2.27
	1-docosahexaenoyl-GPE (22:6)	0.93	0.84
	1-oleoyl-GPE (18:1)	1.29	1.51
	1-palmitoyl-GPC (16:0)	3.16	1.90
Inositol Metabolism	Scyllo-inositol	0.84	0.81
	Myo-inositol	1.12	0.81
Amino Fatty Acids	2-aminoheptanoate	1.38	1.67
	2-aminooctanoate	631.4	9.24*
Fatty Acid Synthesis	2-methylmalonylcarnitine	0.64***	0.69*
Polyunsaturated Fatty Acid	Docosahexaenoate (22:6n3)	2.05	1.07
Primary Bile Acid Metabolism	Glycocholate	12.08*	2.44
Sphingolipid Metabolism	Palmitoyl sphingomyelin (d18:1/16:0)	2.66*	1.73
Corticosteroids	Cortisone	0.73**	1.19
	Cortisol	0.98	1.65
Androgenic Steroid	Androstenediol (3β,17β) disulfate	6.31	6.02
Sterols	7-α-HOCA	0.95	0.88*

Ratios are fold changes from during KD compared to before KD. Compounds are grouped by compound family, and by optimal response ratio within that family. Significant increases are indicated with bold, significant decreases with italics. False-discovery rates: *0.10 < q < 0.20, **0.05 < q < 0.10, ***q < 0.05. 7-HOCA – 7-alpha-hydroxy-3-oxo-4-cholestenoate, CMPF – 3-carboxy-4-methyl-5-propyl-2-furanpropanoate, *GPC* glycerol-3-phosphocholine, *GPE* glycero-3-phosphoethanolamine

CSF lipids and their metabolites were mostly increased by KD treatment (Table 2), including the hydroxylated derivatives and carnitine esters of medium- and short-chain fatty acids. The ketone bodies acetoacetate and β-hydroxybutyrate were increased many-fold, and although the ketone body acetone was not itself detected, its metabolite 1,2-propanediol [35] was detected and elevated significantly. There were large increases in trimethylamine N-oxide (a gut microbiotic metabolite of dietary carnitine and lecithin) and glycocholate (a gut microbiota-modified bile acid). There were decreases in methylmalonyl-carnitine (involved in fatty acid synthesis) and glycerol 3-phosphate (a triglyceride metabolite). Many of these changes were significant only in optimal responders. Regarding compounds that were significantly changed in both patients with an optimal vs. no observable clinical response (e.g. hexanoyl- and acetyl-carnitine, 3-hydroxyoctanoate, trimethylamine N-oxide), examination of the fold changes in means reveals larger magnitude changes in optimal responders.

Not surprisingly, since a KD greatly reduces dietary carbohydrates, CSF sugars and their metabolites/derivatives during KD therapy changed greatly from baseline to during KD (Table 3). Some carbohydrates were decreased, including glucose (significant only in optimal responders) and the glycemic indicator 1,5-anhydroglucitol [36]. A number of hexoses and pentoses, however, were increased. Elevated pyruvate in brain was previously noted in rodents eating a KD [37]. Notably, sorbose and fructose were significantly increased only in patients with no clinical response.

**Table 3:** Diet-induced changes in carbohydrates

		During diet/Prediet	
Compound Family	Compound	optimal response	no response
Glycolysis	1,5-anhydroglucitol	0.57**	0.47**
	Glucose	0.83***	0.93
	Lactate	0.96	1.05
	Glycerate	1.12	0.99
	Pyruvate	1.18***	1.93
Hexose Metabolism	Galactitol (dulcitol)	0.67**	0.87
	Sorbose	0.71*	8.04**
	Sorbitol	1.11	1.27
	Fructose	1.13	1.46**
	Mannose	1.52***	1.35***
	Mannitol	1.66***	1.70**
Pentose Metabolism	Threitol	0.61*	0.74*
	Xylitol	0.94	0.92
	Ribitol	1.04	0.97
	Xylose	1.12	1.04
	Ribose	1.18	1.12
	Ribulose	1.37*	1.16
	Xylonate	1.38**	1.15
	Arabitol	1.54***	1.48**
	Arabinose	2.38***	1.63***
Aminosugar Metabolism	Erythronate	0.84*	0.77**
	N-acetylneuraminate	0.98	0.93
	Glucuronate	1.04	1.04
Advanced Glycation End-product	Erythrulose	1.3	0.58*

Ratios are fold changes from during KD compared to before KD. Compounds are grouped by compound family, and by optimal response ratio within that family. Significant increases are indicated with bold, significant decreases with italics. False-discovery rates: *0.10 < q < 0.20, **0.05 < q < 0.10, ***q < 0.05

As for between-subjects comparisons of CSF metabolites in patients with an optimal vs. no clinical response during KD treatment, no compounds survived application of a false-discovery rate correction. However, several showed differences that were significant with uncorrected t-tests (Table 4), and these demonstrated a pattern that supported the within-subjects comparison. Ketone bodies and some fatty acids and hydroxylated fatty acids were several-fold higher in patients with optimal vs. no clinical response. In addition, glucose, sorbose, and fructose were lower in optimal responders.

**Table 4:** Between-subject differences, optimal v. no response, on and before ketogenic diet

During KD		responders / non-responders
Compound family	Compound	
Ketone Bodies	β-hydroxybutyrate	3.24**
	acetoacetate	3.88**
	1,2-propanediol	4.74*
Monohydroxy Fatty Acids	γ-hydroxybutyrate	2.06**
	3-hydroxyoctanoate	2.61**
Medium Chain Fatty Acid	hexanoic acid	2.79**
Triglyceride Metabolism	glycerol	1.34*
Glycolysis	glycerate	0.62*
	glucose	0.88**
Hexoses	sorbose	0.28**
	fructose	0.75**

Baseline		
Acyl Carnitines	acetylcarnitine	0.72*
Corticosteroids	cortisol	1.36*
Hexoses	mannose	0.72**
Tetroses	erythrulose	0.34*

Ratios are fold difference between patients with an optimal vs. no clinical response. Only compounds with *p* < 0.10 are listed. *0.05 < *p* < 0.10, **p < 0.05

Between-subject comparisons of CSF metabolites in patients with optimal vs. no clinical response in baseline samples revealed only one compound that was significant with an uncorrected t-test at p < 0.05: the hexose mannose, higher in patients with no clinical response (Table 4).

**Fig. 1:**
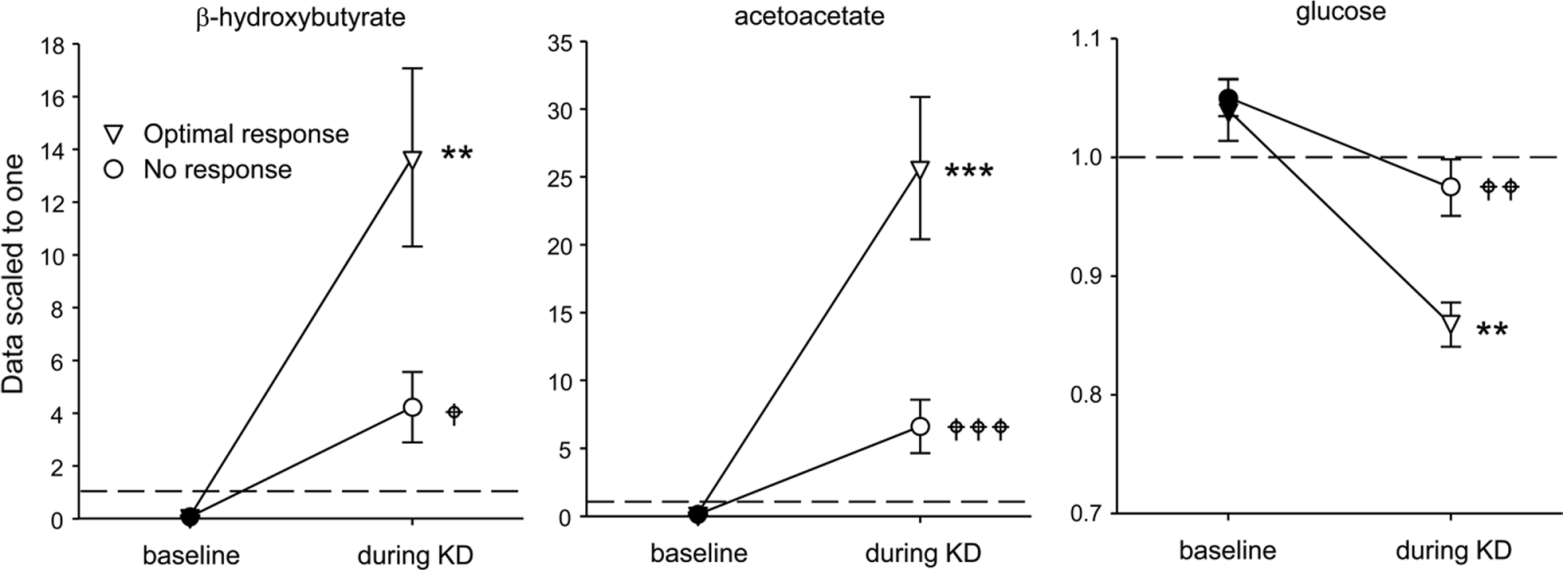
Hallmark changes in ketone bodies β-hydroxybutyrate (left) and acetoacetate (middle) and glucose (right) are greater in CSF from patients with optimal response compared to those with no response. Data are scaled to one (indicated by dashed lines); means and standard errors are shown. See text for quantified β-hydroxybutyrate level. ANOVA group-x-time interactions: β-hydroxybutyrate F = 6.8, *p* = 0.031; acetoacetate F = 11.6, *p* = 0.009; glucose F = 5.8, *p* = 0.043. There were also significant main effects of time and patient group for each metabolite. ** *p* < 0.01, *** *p* < 0.001 compared to pre-KD; ^†^*p* < 0.05, ^††^*p* < 0.01, ^†††^*p* < 0.001 compared to optimal responder during KD

Statistical analysis was further focused on the hallmark changes in biochemistry within the lipid and carbohydrate families, namely the ketone bodies and glucose. We applied two-by-two ANOVAs to these CSF metabolites with clinical response to KD and time as factors. There were no baseline differences between the groups (locants are overlapping in Fig. 1). As expected, ketone bodies (β-hydroxybutyrate and acetoacetate) were elevated and glucose was lowered during KD treatment; however, all these responses were significantly smaller in the patients with no clinical response vs. those with optimal response (Fig. 1). Serum levels of β-hydroxybutyrate during KD were also significantly lower in patients with no clinical response (3.2 ± 0.4 mM) vs. those with optimal response (5.2 ± 0.1 mM, *p* < 0.01), expected as CSF ketone body levels track blood levels [38–42].

Follow-up data are available on all patients. In the 5 patients who rapidly acquired seizure freedom, “optimal responders”, the KD was continued between 2 years and 4.6 years. In three of these the KD was tapered and several years after discontinuation of the diet they were still seizure-free. In two patients, however, seizures came back during tapering: in one there are very few seizures but in the other the seizures were as bad as before KD. In the five patients with no seizure response, the diet was tapered after 3–4 months in three patients, but parents of two patients persevered with the KD because of improved communication and better alertness. Dietary treatment was continued for 2–3 years with these behavioral improvements persisting.

## Discussion

We characterized KD-induced changes in the CSF lipid/carbohydrate metabolome in pediatric epilepsy patients with the most distinct clinical behavioral responses at 3 months: either no detectable seizures (optimal response) or no detectable change in the frequency of refractory seizures. A major finding from this analysis, supported by longitudinal and between-subjects analyses, is that those patients who had an optimal response had a stronger metabolic response to the KD than those patients who showed no seizure improvement. This effect was evident by a larger number of KD-changed compounds and a larger magnitude change in most of those compounds in optimal responders – including glucose and the ketone bodies. While this pattern might be interpreted to indicate that the patients with no clinical response were less compliant, there is some evidence to suggest this is not true. For example, diet compliance was described as good by caretakers and medical personnel. Furthermore, all patients with no clinical response (as well those with optimal response) had striking elevations of CSF ketone bodies during KD compared to pre-diet (and high blood ketone bodies during KD), confirming adherence to a KD that precipitated a shift to ketone-based metabolism. Thus, these differences may be due to inherent biological variability in metabolic response. We cannot rule out, however, that some external dietary factors (exact ratio of KD administered, types of fat or of protein, etc.) could be involved.

Of paramount interest is the identification of compounds that could be mechanistically involved in the anticonvulsant effect of a robust metabolic response. The eponymous products of a KD are an obvious candidate [14]. Beyond being a more efficient fuel for in oxidative metabolism and mitochondrial health, ketone bodies are putative signaling molecules in several pathways that should improve the state of hyperexcitable brain tissue, including inhibitory ion channels [43], vesicular glutamate release [44], inflammation [45], nicotinamide adenine dinucleotide oxidation state [46], and histone deacetylases [47]. It was realized in the earliest KD studies that some threshold level of blood ketone bodies needed to be reached for beneficial outcomes [48, 49]. Beyond that, however, the connection of elevated blood ketone bodies and anticonvulsant effects clinically has been controversial, described as correlated [50–52] or not [53]. Studies have noted that some individual patients benefit despite not being in ketosis [54–56] or have described a correlation only at certain lengths of KD treatment [55, 57].

Besides elevating brain ketone body levels via the liver, low glucose appears to have direct effects in brain. Mildly lowered glucose promotes an autocrine inhibition in pyramidal neurons in vitro based on the neuromodulator adenosine [58]. This same mechanism is invoked by KD treatment [59] and is anti-seizure [60]. Similarly, partial block of glycolysis with 2-deoxyglucose tempers epileptiform activity in vitro [61] and seizures in vivo in some models [62]. Low glycemic index diets can be anticonvulsant in the absence of ketosis [63, 64], and low glucose rather than elevated ketone bodies seems to underlie the anticonvulsant action of caloric restriction [65]. At the other end of the glycolytic pathway, elevated pyruvate was significant in optimal responders only, which may indicate decreased usage due to increased acetyl-CoA production from ketone bodies.

Acetyl-carnitine, associated with dietary red meat [28] is involved in synthesis of acetyl-CoA. Although little work has been done with seizures directly [66], this molecule is thought to be neuroprotective, neurotrophic, and antioxidant. Notably, acetyl-carnitine is well-tolerated as a dietary supplement [67].

Conversely, this metabolomics analysis revealed some elevated compounds that may be mechanistically detrimental to the efficacy of a KD. KD-related elevations in fructose and sorbose occur in patients with no clinical response and not those with optimal response. Because fructose and sorbose can be metabolized to fructose-6-phosphate, these KD-related changes could augment glycolytic metabolites downstream of glucose. Combined with a smaller reduction in glucose itself in patients with no clinical response, this pattern might indicate that substantial amounts of acetyl-coA are still entering the Kreb’s cycle from the glycolytic pathway. This state may offset the enhanced efficiency of adenosine triphosphate production during ketolysis, undermining the beneficial effects of the KD-related metabolic state presumably occurring with full force in optimal responders.

Many detected compounds likely reflect a stronger metabolic response to the KD in optimal responders without being mechanistically involved in a better anticonvulsant response. For instance, increases in trimethylamine N-oxide is to be expected in a diet rich in meat, fish, and eggs; changes in glycocholate and methylmalonyl carnitine comport with a high fat diet; decreased glycerol 3-phosphate could be due to increased shunting into glycolysis (via dihydroxyacetone phosphate) to augment this underpowered pathway – none of these metabolites are thought to have signaling properties in brain. 3-hydroxyoctanoate is a signaling molecule with specific receptors expressed in adipocytes but not brain [68]. γ-hydroxybutyrate also acts on specific receptors (GPR172A), and is used exogenously as an abused hypnotic/sedative drug. However, antagonism of GPR172A is anticonvulsant; conversely, activation is used to produce an animal model of absence seizures [69] making higher γ-hydroxybutyrate unlikely to be involved in a better anticonvulsant response to the KD. It was hypothesized that elevated β-hydroxybutyrate might mediate the mild euphoria found during fasting and sometimes during KD [70] by acting pharmacologically like γ-hydroxybutyrate; however, elevated γ-hydroxybutyrate itself may underlie this effect.

These findings can be related to prior clinical studies of the plasma metabolome, and some changes found in plasma were not present in these CSF samples. For instance, KD did not elevate cortisol in CSF, whereas it has been reported to do so in plasma; these latter studies were, however, in rheumatoid arthritis patients [19, 53]. Cappuccio et al. reported elevated plasma hydroxybutyryl- and acetyl-carnitine and lower methylmalonyl-carnitine in KD-treated glucose transporter 1 deficient patients [16], similar to the present results. Also, Schoeler et al. demonstrated a stronger β-hydroxybutyrate response to KD treatment in patients with optimal clinical response vs. those with no response; however, they also reported that optimal responders had higher baseline CSF β-hydroxybutyrate and octanoyl- and acetyl-carnitine [21]. We did not replicate those findings, and in fact showed a trend for lower initial acetyl-carnitine in optimal responders (and a significant increase during KD was also specific to optimal responders – showing a strong regulation by KD). These discrepancies could be due to true differences between plasma and CSF, or might reflect dissimilar definitions of patients with optimal response or no clinical response.

We acknowledge some limitations to the present study. The number of subjects is low, which reduces statistical power. Also, we are unaware of any clinical study that describes effects of the presently-used anticonvulsants on CSF levels of our metabolites of interest, so if and how these drugs had any effect remains unknown. Last, metabolomics was not performed on plasma, which was collected but lost in a freezer accident. This could have indicated correlations between CSF and plasma and possibly identified blood-born predictors of clinical response.

## Conclusions

In a small but valuable clinical cohort with pre- and post-KD CSF sampling, we found a more effective anticonvulsant response correlated with a stronger central metabolic response at 3 months of KD treatment. Such samples present a special opportunity as studies with sequential lumbar punctures in pediatric patients are rare, but allow for within-subjects analysis. This study extends and complements prior work on monoamine and amino acid neurotransmitters in CSF [22, 23]. Focused studies of specific metabolites are valuable, but the unpredicted changes found here (such as KD-elevated levels of hexoses only in non-reponders) highlight the importance of casting a wide net. Further work is needed and could determine more quantitatively if a strong central metabolic response early in KD treatment predicts an ultimately effective anticonvulsant outcome, information that may be useful in decisions about treatment adjuvants, adjustments and/or continuation. In addition, a blood-based correlate that predicts efficacy would be extremely valuable because CSF collection is necessarily invasive. It is worth noting that some of the relevant metabolites (ketone bodies, 1,2-propanediol, glycerol 3-phosphate) are detectable with ^1^H or ^31^P nuclear magnetic resonance spectroscopy. For those whose poor anticonvulsant response may due to inherent biological factors, on possibility is that KD treatment could be amplified by manipulating hallmark metabolic changes directly – for example supplementing with relevant molecules, e.g. ketone bodies (via precursors such as ketone esters, medium chain triglycerides, or coconut oil) or acetyl-carnitine.

## Data Availability

The dataset produced and analyzed in this study is available from the corresponding author on reasonable request.

## Abbreviations

7-HOCA: 7-alpha-hydroxy-3-oxo-4-cholestenoate
AED: Antiepileptic drug
CLB: Clobazam
CMPF: 3-carboxy-4-methyl-5-propyl-2-furanpropanoate
CSF: Cerebrospinal fluid
CZP: Clonazepam
GC-MS: Gas chromatography–mass spectrometry
GPC: Glycero-3-phosphocholine
GPE: Glycero-3-phosphoethanolamine
HESI: Heated electrospray ionization
KD: Ketogenic diet
L-G: Lennox-Gastaux syndrome
LTG: Lamotrigine
PB: Phenobarbital
PRI: Primidone
T/C: Tonic/clonic
TPM: Topiramate
UPLC-MS/MS: Ultra-high performance liquid chromatography-tandem mass spectrometry
VPA: Valproic acid
